# An ultrasound-based nomogram for predicting early neonatal necrotizing enterocolitis

**DOI:** 10.3389/fped.2026.1907406

**Published:** 2026-10-20

**Authors:** Junxian RUAN, Huaiying SU, Ying HE, Weiru LIN

**Affiliations:** 1 Ultrasound Department, Quanzhou Maternal and Child Health Hospital (Quanzhou Children’s Hospital), Quanzhou, Fujian, China; 2 Imaging Department, Quanzhou Maternal and Child Health Hospital (Quanzhou Children’s Hospital), Quanzhou, Fujian, China; 3 Neonatology Department, Quanzhou Maternal and Child Health Hospital (Quanzhou Children’s Hospital), Quanzhou, Fujian, China

**Keywords:** neonatal necrotizing enterocolitis, nomogram, prediction model, preterm infant, risk factors, ultrasound

## Abstract

**Objective:**

To develop and validate an ultrasound-based nomogram prediction model for the early diagnosis of neonatal necrotizing enterocolitis (NEC).

**Methods:**

This is a retrospective cohort study. A total of 500 preterm infants admitted to the neonatal intensive care unit (NICU) of our hospital from January 2023 to June 2024 were enrolled. They were stratified by NEC status and randomly assigned to a development cohort and a validation cohort at a ratio of 7:3. According to whether NEC occurred, infants were further categorized into an NEC group and a non-NEC group. General clinical data, including gestational age and birth weight, were collected. Laboratory inflammatory markers included C-reactive protein (CRP), procalcitonin (PCT), and interleukin-6 (IL-6). Ultrasound parameters included bowel wall thickness, intestinal peristalsis, gastric antral area, gastric antral peristalsis, and bowel wall blood perfusion. Logistic regression analysis was performed in the development cohort to identify independent risk factors and construct a nomogram prediction model. Receiver operating characteristic (ROC) curves, calibration curves, and decision curve analysis were used to evaluate model discrimination, calibration, and clinical net benefit in the development cohort. The validation cohort was used to verify model performance.

**Results:**

Univariate analysis in the development cohort showed that gestational age, birth weight, CRP, PCT, IL-6, bowel wall thickness, intestinal peristalsis, gastric antral area, gastric antral peristalsis, and bowel wall blood perfusion differed significantly between groups (*P* < 0.05). Multivariable logistic regression further identified gestational age, CRP, PCT, IL-6, bowel wall thickness, intestinal peristalsis, bowel wall blood perfusion, gastric antral area, and gastric antral peristalsis as independent factors associated with early NEC (*P* < 0.05). On this basis, a nomogram prediction model was established. The areas under the ROC curve (AUCs) in the development and validation cohorts were 0.88 and 0.85, respectively, indicating good discriminative ability. The optimal risk cut-off of 0.18 was determined by Youden index solely from the development cohort. At this fixed threshold, sensitivity, specificity, PPV and NPV were 82.4%, 81.2%, 51.4%, and 95.0% for the development cohort; the corresponding performance in the validation cohort was 77.2%, 76.0%, 43.8%, and 92.4%. Goodness-of-fit testing of the calibration curves showed chi-square = 1.235 and *P* = 0.998 in the development cohort, and chi-square = 1.642 and *P* = 0.995 in the validation cohort, suggesting favorable agreement between predicted probabilities and observed risks. Decision curve analysis showed that when the risk threshold was greater than 0.05, the nomogram provided significant clinical net benefit.

**Conclusion:**

The nomogram prediction model constructed from inflammatory and multiparametric ultrasound indicators demonstrates favorable predictive performance for the early diagnosis of neonatal NEC. It may provide clinicians with a noninvasive and repeatable auxiliary diagnostic tool, facilitating early intervention for NEC and improving prognosis.

## 1 Introduction

Neonatal necrotizing enterocolitis (NEC) is the most common severe gastrointestinal emergency among preterm and very-low-birth-weight infants. It is characterized pathologically by bowel wall ischemia, inflammation, and necrosis. The disease has an insidious onset and progresses rapidly, with a clinical case fatality rate of 20% to 50%. Survivors often develop long-term complications such as intestinal stricture and short bowel syndrome, which substantially impair quality of life (1, 2).

At present, the clinical diagnosis of NEC is mainly based on Bell staging criteria in combination with clinical manifestations, imaging findings, and laboratory indicators. However, early symptoms lack specificity, and conventional abdominal radiography is often unable to detect subtle early bowel wall lesions, which may lead to missed diagnosis and diagnostic delay (3, 4).

Abdominal ultrasound is noninvasive, repeatable, and radiation-free. It can clearly display bowel wall thickness, peristaltic function, blood perfusion, and subtle intra-abdominal changes. Therefore, ultrasound can capture early pathophysiological alterations of the bowel wall in NEC and provide imaging support for early recognition (5, 6).

A nomogram is a visual prediction tool that converts multivariable regression results into an intuitive scoring system. It allows clinicians to rapidly quantify the risk of disease occurrence and has been widely applied in risk prediction across multiple diseases (7, 8). Existing NEC prediction models generally rely on laboratory indicators or ultrasound indicators alone, and an ultrasound-based nomogram that integrates both types of indicators and undergoes validation has not yet been fully established.

In this study, preterm infants admitted to the NICU were enrolled. Laboratory inflammatory indicators and multiparametric ultrasound findings were integrated, independent risk factors for early NEC were screened using multivariable logistic regression, and an ultrasound-based nomogram prediction model was constructed. The model was validated using development and validation cohorts to provide a noninvasive quantitative diagnostic tool for early NEC recognition and early intervention, with the aim of reducing mortality and improving outcomes.

## 2 Materials and methods

### 2.1 Study population

This retrospective cohort study included 500 preterm infants admitted to the NICU of our hospital between January 2023 and June 2024. Stratified random sampling based on NEC occurrence status was adopted to maintain consistent NEC prevalence across sub-cohorts. Using a random number table, infants were assigned at a ratio of 7:3 to a development cohort (*n* = 350) and a validation cohort (*n* = 150). In each cohort, infants were further divided into an NEC group and a non-NEC group according to whether NEC occurred during hospitalization. In the development cohort, 68 infants developed NEC and 282 did not. In the validation cohort, 29 infants developed NEC and 121 did not.

The inclusion criteria were as follows: (1) preterm infants with gestational age < 37 weeks; (2) admission to the NICU within 24 h after birth; (3) complete clinical, laboratory, and ultrasound data; and (4) written informed consent provided by the infant's family.

The exclusion criteria were as follows: (1) congenital gastrointestinal malformations, congenital heart disease, or inherited metabolic diseases; (2) severe asphyxia at birth or severe intracranial hemorrhage; (3) surgical treatment after admission; and (4) hospitalization for < 72 h or loss to follow-up.

The diagnosis of NEC was based on the modified Bell staging criteria for neonatal NEC (9, 10). In this study, all infants in the NEC group had early-stage disease, classified as stage I to II; severe stage III cases were excluded. Sample size was estimated based on the events-per-variable (EPV) principle for logistic regression, requiring at least 10 events per predictor variable. This nomogram contained 9 independent predictors; thus a minimum of 90 NEC events was required. Assuming an approximate 20% incidence of early-stage NEC among preterm infants and a 0% loss-to-follow-up rate according to our preliminary clinical data, at least 450 subjects were needed. To accommodate the 7:3 split for model development and validation and to improve model stability, we increased the total sample size to 500 preterm infants. The study was approved by the Medical Ethics Committee of Quanzhou Maternal and Child Health Hospital (Quanzhou Children's Hospital) (Ethical Approval Number: 2022 Lun Shen No.54). Written informed consent was obtained from the families of all enrolled infants, permitting the use of clinical and examination data for research purposes.

### 2.2 Methods

#### 2.2.1 Data collection

General information, including gestational age, birth weight, sex, mode of delivery, and Apgar scores, was collected through the hospital electronic medical record system. Peripheral venous blood was collected within 72 h after admission. CRP was measured using an automated biochemical analyzer; PCT was measured by electrochemiluminescence; and IL-6 was measured by enzyme-linked immunosorbent assay (ELISA). The results of each indicator were recorded.

Within 72 h after admission, abdominal ultrasound was performed by two senior ultrasonographers using a color Doppler ultrasound diagnostic system with a probe frequency of 7.5 to 10.0 MHz. Ultrasonographers were blinded to patients' clinical manifestations and laboratory results during ultrasound acquisition. Inter-rater reliability was evaluated in 80 randomly selected study cases that were independently assessed by both operators. The intraclass correlation coefficient (ICC) for bowel wall thickness was 0.84(95%CI: 0.75–0.90), and ICC for gastric antral area was 0.81(95%CI: 0.71–0.88). For categorical ultrasound parameters, Cohen's kappa was 0.78 for intestinal peristalsis, 0.80 for bowel wall blood perfusion, and 0.76 for gastric antral peristalsis. Bowel wall thickness, intestinal peristalsis, gastric antral area, gastric antral peristalsis, and bowel wall blood perfusion were measured and recorded.

The ultrasound diagnostic criteria were as follows (11): bowel wall thickness ≥ 2 mm was defined as thickening; intestinal peristalsis < 3 times/min was defined as decreased intestinal peristalsis; gastric antral area > 1.5 cm^2^ was defined as enlargement; gastric antral peristalsis < 2 times/min was defined as decreased gastric antral peristalsis. According to the grading standards of previous study (12), the intestinal wall blood perfusion was semi-quantitatively graded: (1) normal perfusion: 1–9 color Doppler signal points per square centimeter of intestinal wall; (2) sparse perfusion: less than 1 color point per cm^2^, residual Scattered weak mural signals; (3) lack of perfusion: complete disappearance of intramural color Doppler signals; (4) overperfusion: >9 color dots per square centimeter or characteristic congestion pattern, including zebra-shaped, ring-shaped or Y-shaped vessel wall signals. Scarcity, absence, or overperfusion of blood flow in the intestinal wall are collectively referred to as abnormal blood perfusion. Sparse, absent, or hyperperfused bowel wall blood flow was defined as abnormal blood perfusion.

#### 2.2.2 Follow-up procedures

All infants were followed for 28 days after admission. Clinical symptoms, physical signs, and laboratory indicators were monitored daily. The occurrence of NEC was determined based on clinical findings combined with abdominal ultrasound and radiographic examination. All 500 infants completed follow-up, corresponding to a follow-up rate of 100%.

### 2.3 Statistical analysis

Statistical analyses were performed using SPSS 26.0 and MedCalc 19.2. The Shapiro–Wilk test was applied to assess normality for continuous variables. Normally distributed continuous variables were expressed as mean ± standard deviation and compared between groups using the independent-samples t test. Apgar scores are ordinal non-normal variables, hence the Mann–Whitney U test was used for comparison. Nonnormally distributed continuous variables were expressed as median (interquartile range) [M (P25, P75)] and compared using the Mann–Whitney U test. Categorical variables were expressed as frequency (%) and compared using the chi-square test or Fisher's exact test.

Before splitting, all enrolled infants were stratified according to whether early NEC occurred. Within each stratum, each subject was allocated a unique random number generated by a random number table. Subjects within each stratum were then divided into development and validation sets at a 7:3 ratio, which guaranteed similar NEC event rates between the two cohorts. Univariate analysis was used to screen potential risk factors for early NEC. Variables with *P* < 0.05 were included in multivariable logistic regression analysis to identify independent risk factors and construct the nomogram prediction model. Variance inflation factor (VIF) was calculated to assess multicollinearity; VIF > 5 was defined as indicative of substantial collinearity. Sensitivity analyses with sequential single-inflammatory-marker removal were conducted to evaluate the robustness of regression coefficient estimates. ROC curves were used to calculate the AUC and evaluate model discrimination. Calibration curves were used to assess agreement between predicted probabilities and observed event rates. Decision curve analysis was used to evaluate the clinical net benefit of the model. *P* < 0.05 was considered statistically significant.

## 3 Results

### 3.1 Univariate analysis of factors associated with early neonatal NEC

Comparison of clinical data between the two groups showed no statistically significant differences in sex, mode of delivery, 1-minute Apgar score, or 5-minute Apgar score between the NEC and non-NEC groups (*P* > 0.05). In contrast, gestational age, birth weight, CRP, PCT, IL-6, bowel wall thickness, intestinal peristalsis, gastric antral area, gastric antral peristalsis, and bowel wall blood perfusion all differed significantly between the two groups (*P* < 0.05). See Table 1 for details.

**Table 1:** Univariate analysis of early neonatal NEC occurrence in the development cohort.

Index	Occurrence group (*n* = 68)	No occurrences (*n* = 282)	t/*χ*^2^/Z	*P*
Gender (e.g., %)			0.071	0.790
male	39 (57.35)	162 (57.45)	-	-
female	29 (42.65)	120 (42.55)	-	-
Gestational age (weeks, $\bar{x}\pm s$)	31.17 ± 2.21	34.94 ± 2.03	-12.741	<0.001
Birth weight (g $\bar{x}\pm s$)	1,442.74 ± 364.21	2,197.43 ± 428.17	-13.805	<0.001
Method of delivery (e.g., %)			0.204	0.651
vaginal delivery	31 (45.59)	134 (47.52)	-	-
Cesarean section	37 (54.41)	148 (52.48)	-	-
1-minute Apgar score [points, M (P25, P75)]	7 (6, 8)	7 (6, 8)	0.307	0.759
5-minute Apgar score [points, M (P25, P75)]	8 (7, 9)	8 (7, 9)	0.163	0.871
CRP (mg/L $\bar{x}\pm s$)	18.81 ± 5.47	6.14 ± 2.20	18.447	<0.001
PCT (ng/mL, $\bar{x}\pm s$)	3.94 ± 1.31	0.82 ± 0.33	22.913	<0.001
IL-6 (pg/mL $\bar{x}\pm s$)	158.74 ± 47.13	57.41 ± 18.62	19.706	<0.001
Intestinal wall thickness ≥2 mm (e.g., %)	62 (91.18)	73 (25.89)	74.803	<0.001
Decreased intestinal motility (e.g., %)	60 (88.24)	62 (21.99)	66.421	<0.001
Gastric antrum area > 1.5 cm^2^ (examples, %)	57 (83.82)	67 (23.76)	61.485	<0.001
Decreased antral motility (e.g., %)	55 (80.88)	60 (21.28)	57.227	<0.001
Abnormal intestinal wall blood perfusion (cases, %)	64 (94.12)	54 (19.15)	84.017	<0.001

### 3.2 Multivariable logistic regression analysis of early neonatal NEC

Variables with *P* < 0.05 in the univariate analysis were entered as independent variables, and the occurrence of early NEC was used as the dependent variable for multivariable logistic regression analysis. Although birth weight was significant in univariate analysis, it exhibited strong collinearity with gestational age. Collinearity diagnostics yielded VIF = 7.82 and tolerance = 0.128 for gestational age, and VIF = 8.14 and tolerance = 0.123 for birth weight. Given substantial multicollinearity, birth weight was excluded from the final multivariable model, retaining gestational age for subsequent modelling.

Collinearity diagnostics were further performed for all nine candidate predictors retained in the final model. The VIF values were as follows: gestational age = 1.31; CRP = 1.42; PCT = 1.48; IL-6 = 1.36; bowel wall thickness = 1.22; intestinal peristalsis = 1.28; gastric antral area = 1.24; gastric antral peristalsis = 1.21; bowel wall blood perfusion = 1.33. All VIF values were below the conventional threshold of 5, indicating no meaningful multicollinearity among the final-model predictors.

To test model robustness, sensitivity analyses were performed by sequentially removing one inflammatory marker (CRP, PCT, or IL-6) at a time and refitting the multivariable logistic regression. After each single-variable exclusion, the direction and statistical significance of remaining predictors' odds ratios remained unchanged, and the development-cohort AUC only fluctuated within a narrow range (0.861-0.883). These findings confirmed the stability of regression outputs.

The results showed that gestational age, CRP, PCT, IL-6, bowel wall thickness, decreased intestinal peristalsis, enlarged gastric antral area, decreased gastric antral peristalsis, and abnormal bowel wall blood perfusion were independent predictors of early neonatal NEC (*P* < 0.05). For details, please refer to Tables 2 and 3.

**Table 2:** Variable assignment table for multivariate logistic regression analysis.

Variable name	Assignment
Early NEC occurrence state	Occurred = 1, Not Occurred = 0
gestational age	Input the original value (week).
CRP	Enter the original value (mg/L)
PCT	Input original value (ng/mL)
IL-6	Enter the original value (pg/mL)
Intestinal wall thickness	≥2mm = 1, <2mm = 0
Intestinal peristalsis	Reduce = 1, Normal = 0
Gastric antrum area	>1.5cm^2^ = 1, ≤1.5cm^2^ = 0
Gastric antral motility	Reduce = 1, Normal = 0
Intestinal wall blood perfusion	Abnormal = 1, Normal = 0

**Table 3:** Multivariate logistic regression analysis of early neonatal NEC.

Variable	*β*	SE	Wald	*P*	OR	95% CI
gestational age	−0.185	0.032	33.066	<0.001	0.831	0.765–0.902
CRP	0.217	0.047	21.368	<0.001	1.242	1.136–1.358
PCT	0.326	0.052	39.018	<0.001	1.385	1.259–1.533
IL-6	0.198	0.042	22.369	<0.001	1.219	1.127–1.318
Intestinal wall thickness ≥2mm	0.452	0.058	60.025	<0.001	1.572	1.415–1.748
Decreased intestinal peristalsis	2.106	0.325	41.896	<0.001	8.215	4.369–15.447
Gastric antrum area > 1.5 cm^2^	0.897	0.145	38.965	<0.001	2.452	1.896–3.168
Decreased gastric antral motility	1.983	0.332	35.789	<0.001	7.265	3.892–13.568
Abnormal intestinal wall blood perfusion	2.674	0.318	71.026	<0.001	14.456	7.892–26.478
constant	-8.965	1.025	76.892	<0.001	0.000	-

OR is the odds ratio; CI is the confidence interval; β is the regression coefficient; SE is the standard error.

### 3.3 Construction of the ultrasound-based nomogram prediction model for early neonatal NEC

Based on the multivariable logistic regression results, an ultrasound-based nomogram prediction model for early neonatal NEC was constructed using nine independent risk factors as predictors: gestational age, CRP, PCT, IL-6, bowel wall thickness, intestinal peristalsis, gastric antral area, gastric antral peristalsis, and bowel wall blood perfusion. The nomogram included a scoring axis for each predictor, a total score axis, and a risk axis for early NEC. Clinicians can assign points according to the infant's actual values for each indicator, sum the points to obtain a total score, and directly estimate the probability of early NEC. See Figure 1.

**Figure 1:**
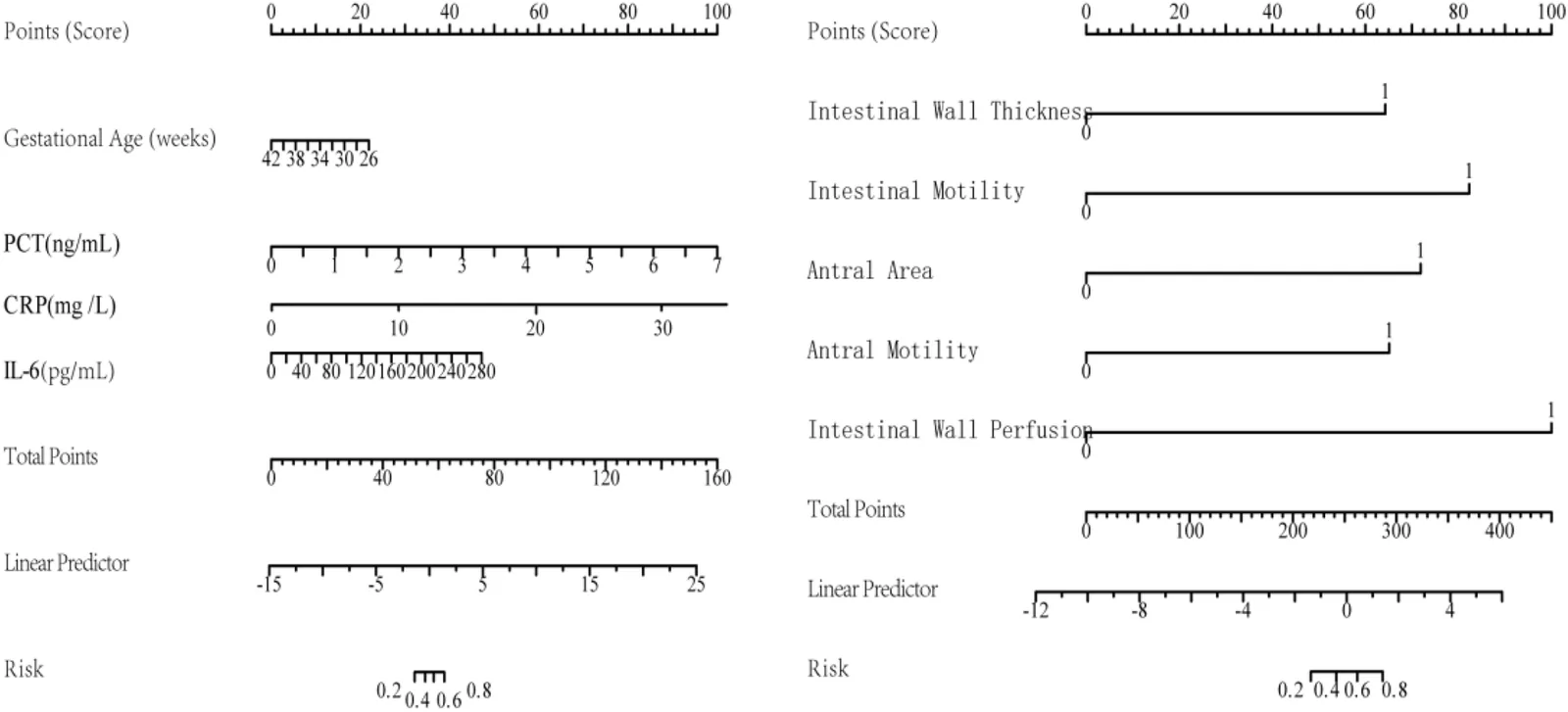
Ultrasound-based nomogram prediction model for early neonatal NEC. To calculate predicted NEC risk: obtain points for each predictor from the top-most Points axis according to each infant's actual indicator values; sum all individual points to get the total points; map the total-points value onto the Risk axis to read the corresponding probability of early NEC.

### 3.4 Calibration curve and clinical net benefit analysis of the prediction model

Goodness-of-fit testing of the calibration curves showed that the calibration curves of both the development and validation cohorts were close to the ideal curve, indicating good agreement between the predicted probability and the observed probability of early NEC. In the development cohort, chi-square = 1.235 and *P* = 0.998; in the validation cohort, chi-square = 1.642 and *P* = 0.995. Neither result showed a statistically significant difference, supporting favorable calibration.

Decision curve analysis showed that when the predicted risk threshold for early NEC was > 0.05 in the development cohort and > 0.05 in the validation cohort, the net benefit of the nomogram prediction model was significantly higher than that of the “treat-all” and “treat-none” strategies. These findings indicate that the model can provide meaningful clinical net benefit within this threshold range. See Figure 2 and Figure 3.

**Figure 2:**
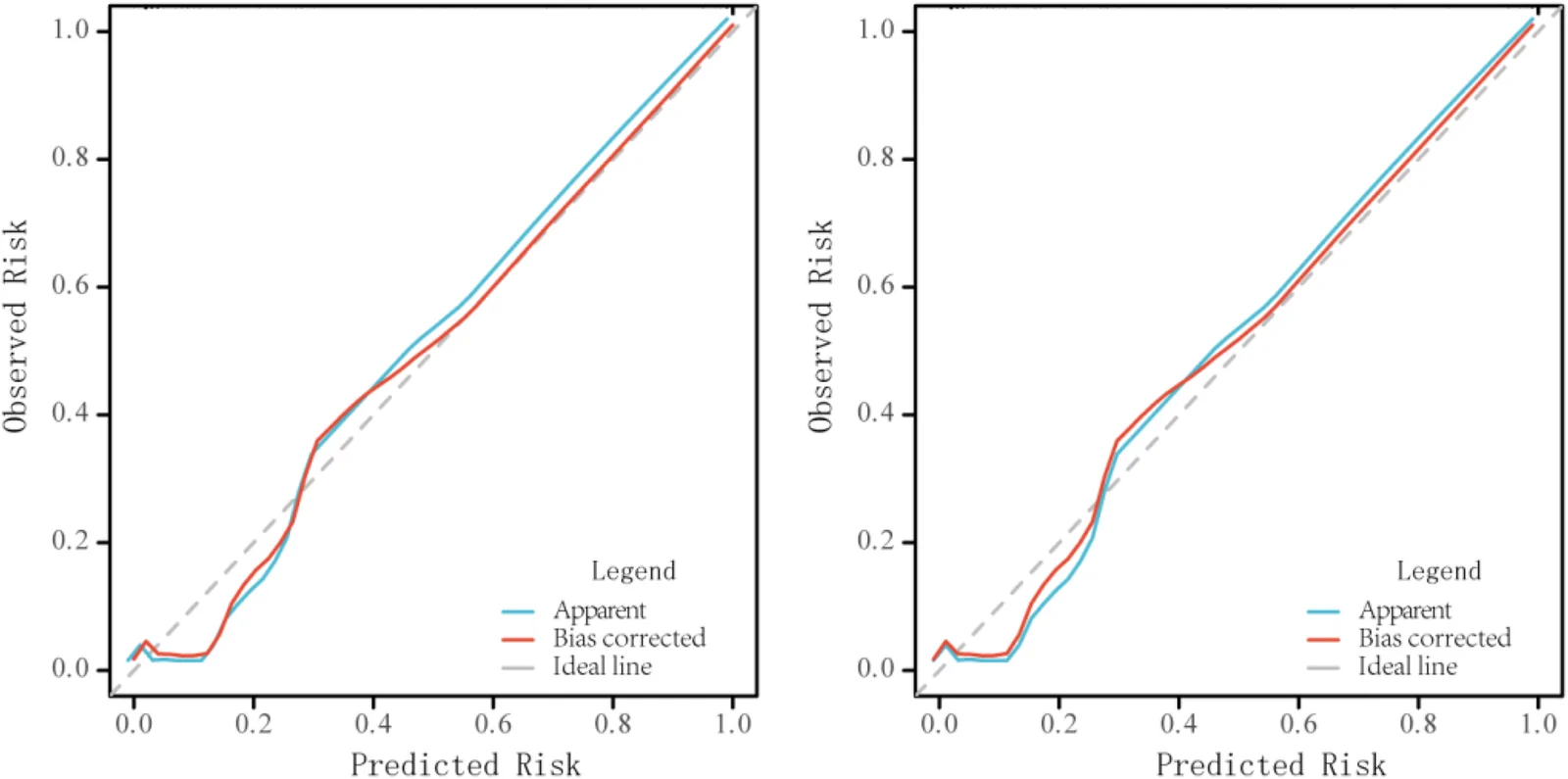
Calibration curves for the ultrasound-based nomogram prediction model. Left: Development cohort; Right: Validation cohort. The blue “Apparent” curve represents uncorrected prediction performance; red “Bias corrected” curve represents bias-corrected performance; grey dashed ideal line represents perfect agreement between predicted and observed NEC risk.

**Figure 3:**
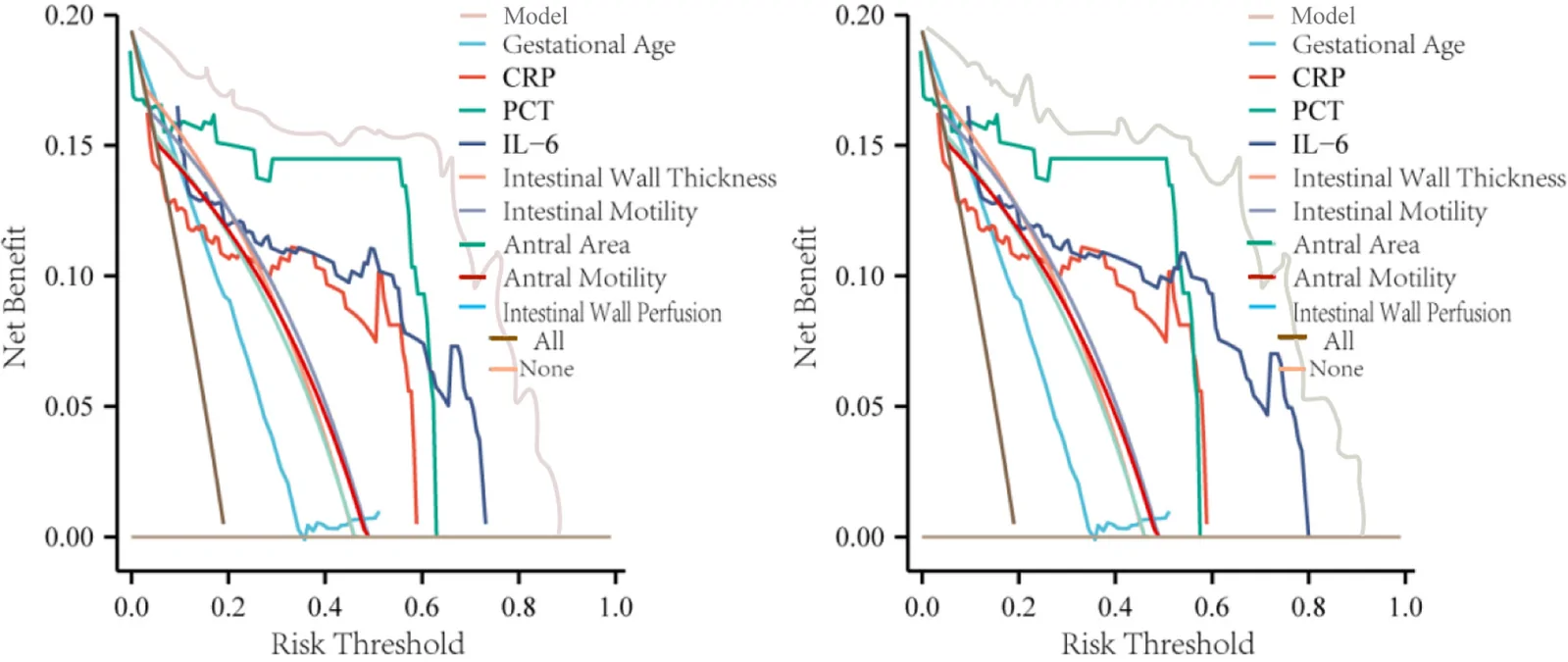
Decision-curve analysis for the ultrasound-based nomogram prediction model. Left: Development cohort; Right: Validation cohort.

### 3.5 ROC curve analysis of the prediction model

ROC curve analysis showed that the AUC of the early NEC prediction model was 0.880 (95% CI: 0.845–0.912) in the development cohort and 0.850 (95% CI: 0.796–0.898) in the validation cohort. Using the Youden-index criterion to determine the optimal risk cut-off value, the optimal threshold was 0.18 for the development cohort, with sensitivity of 82.4%, specificity of 81.2%, positive predictive value of 51.4%, and negative predictive value of 95.0%. This identical cut-off of 0.18 was then applied to the independent validation cohort, giving sensitivity 77.2%, specificity 76.0%, PPV 43.8%, and NPV 92.4%. The slightly lower PPV in the validation cohort, despite similar NEC prevalence, may reflect modest overfitting or sampling variability, and warrants attention in future external validation studies. These results suggest that the ultrasound-based nomogram prediction model has good discriminative ability for early neonatal NEC and can effectively distinguish infants at higher versus lower risk of NEC. See Figure 4.

**Figure 4:**
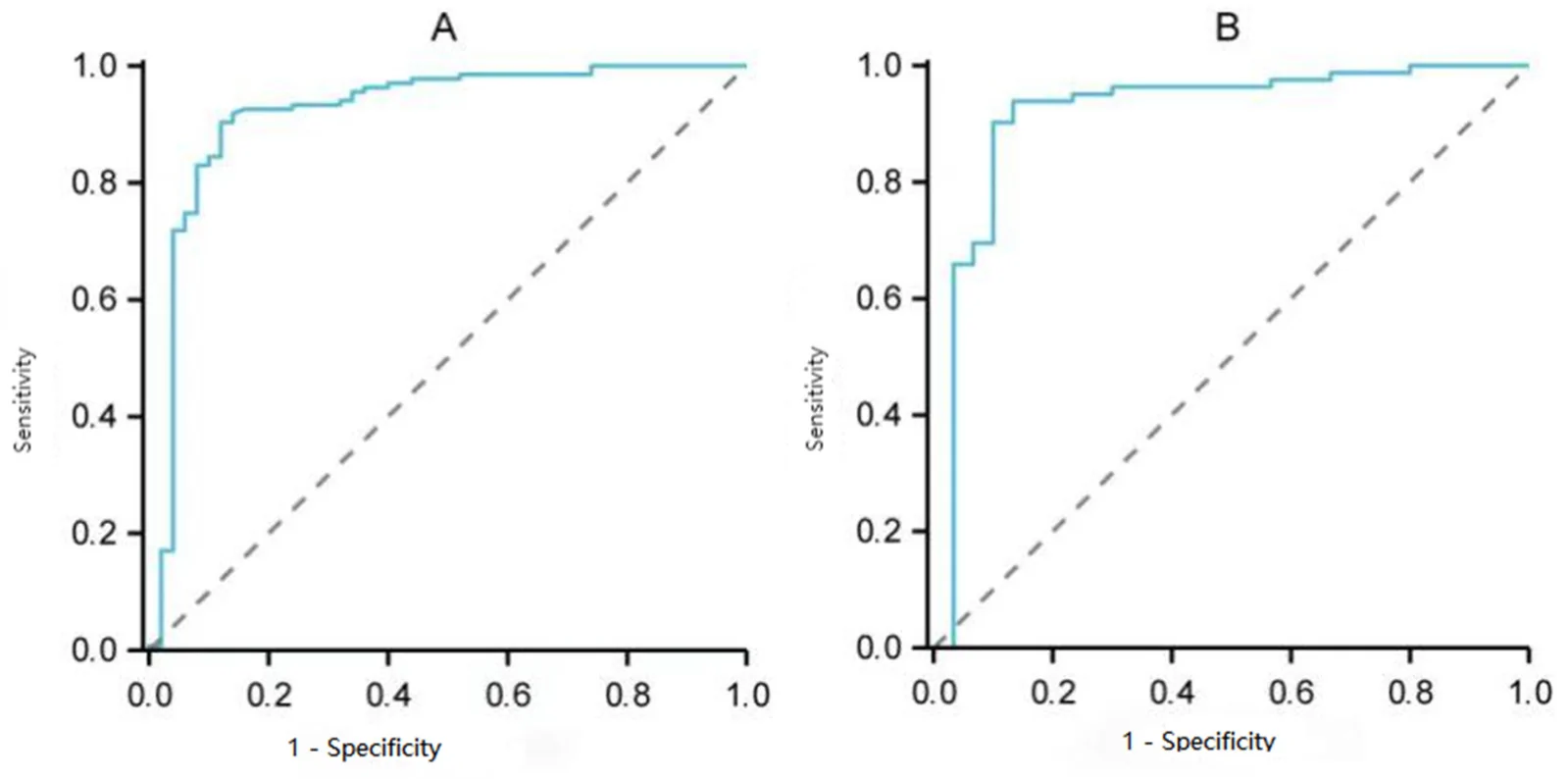
ROC curves of the ultrasound-based nomogram prediction model **(A)** development cohort; **(B)** validation cohort.

## 4 Discussion

In this study, 500 preterm infants were enrolled and divided at a ratio of 7:3 into a development cohort and a validation cohort. Through univariate analysis and multivariable logistic regression, gestational age, CRP, PCT, IL-6, and multiparametric ultrasound indicators were identified as independent predictors of early neonatal NEC. The ultrasound-based nomogram prediction model constructed from these nine indicators demonstrated favorable predictive performance in both cohorts. The AUC values in the development and validation cohorts were 0.880 and 0.850, respectively. The calibration curves were highly consistent with the ideal curve, and the model showed significant clinical net benefit when the risk threshold was > 0.05. These results suggest that the model may provide a reliable quantitative tool for the early, noninvasive diagnosis of NEC.

The present study showed that gestational age was a protective factor for early NEC (OR = 0.831), indicating that the smaller the gestational age, the higher the risk of NEC. This finding is closely related to the pathophysiological characteristics of intestinal immaturity in preterm infants (13). In preterm infants with gestational age < 34 weeks, the intestinal mucosal barrier is not fully developed, intercellular junctions between intestinal epithelial cells are loose, the intestinal microbiota is prone to translocation, and intestinal motility is poor. Prolonged retention of food in the intestine can easily trigger intestinal inflammatory responses and thereby induce NEC.

CRP, PCT, and IL-6 are classic inflammatory markers that increase significantly during the early stages of NEC and directly reflect intestinal inflammatory activity (14). The pathogenesis of NEC is closely associated with excessive intestinal inflammation. Intestinal ischemia-hypoxia and bacterial translocation can activate inflammatory cells and induce the release of large quantities of inflammatory mediators, creating an inflammatory cascade that results in bowel wall injury and necrosis. CRP, PCT, and IL-6 sensitively reflect the degree of intestinal inflammation; higher levels indicate more severe inflammatory activity and a higher risk of NEC.

Multiparametric ultrasound indicators have important clinical value in the early diagnosis of NEC. In this study, bowel wall thickening, decreased intestinal peristalsis, enlarged gastric antral area, decreased gastric antral peristalsis, and abnormal bowel wall blood perfusion were all independent predictors of early NEC. Among them, abnormal bowel wall blood perfusion had an OR value of 14.456, making it the most critical ultrasound indicator affecting NEC occurrence.

Bowel wall thickening directly reflects intestinal inflammation and edema. In the early stage of NEC, congestion and edema of the intestinal mucosa and submucosa can increase bowel wall thickness (15). Decreased intestinal and gastric antral peristalsis is associated with immature enteric nervous system development and inflammation-mediated inhibition of intestinal smooth muscle contraction. Slowed gastrointestinal motility further aggravates intestinal stasis, promotes bacterial proliferation, and intensifies inflammatory responses (16).

Abnormal bowel wall blood perfusion is an important pathological change in early NEC. Intestinal ischemia and hypoxia are core elements in NEC pathogenesis. Factors such as umbilical arterial catheterization and inappropriate feeding may reduce intestinal blood flow, resulting in low or absent perfusion of the bowel wall. As inflammation worsens, hyperperfusion of the bowel wall may occur, leading to ischemia-reperfusion injury and further aggravating bowel wall necrosis (17). Abdominal ultrasound can clearly identify these subtle bowel wall lesions, thereby providing imaging evidence for early NEC diagnosis and compensating for the limitations of conventional radiography.

The findings of this study are consistent with several recent studies and also highlight its distinct innovative value. For example, Garg et al. (18) reported in a study of NEC in preterm infants that CRP, PCT, IL-6, bowel wall thickness, and abnormal bowel wall blood perfusion were independent predictors of NEC, which is consistent with the present findings and further supports the value of combining inflammatory markers with ultrasound indicators for early NEC diagnosis. Kim et al. (19) highlighted the high risk of NEC among extremely preterm infants; El Manouni El Hassani et al. (20) demonstrated that inflammatory biomarkers and intestinal ultrasound manifestations are closely correlated with NEC severity. Yu et al. (21) also reported elevated inflammatory indices and abnormal intestinal sonographic features in preterm infants with early-onset NEC. Collectively, these studies further support the clinical value of integrating inflammatory markers and multiparametric ultrasound indicators for the early identification of early-stage NEC.

Compared with previous studies, several published nomograms have been constructed for predicting neonatal NEC; most of these models rely exclusively on clinical-laboratory variables without incorporating quantitative abdominal ultrasound metrics, while a small number of ultrasound-focused predictive tools use only isolated sonographic findings. The innovation of this study lies in several aspects. First, laboratory inflammatory indicators and multiparametric ultrasound indicators were integrated to establish a multidimensional predictive model, avoiding the limitations of diagnosis based on a single indicator. Second, the nomogram visualized complex regression results, allowing clinicians to rapidly calculate an infant's NEC risk through simple point assignment and summation. This approach is convenient and more suitable for implementation in routine NICU practice. Third, the model was validated in both the development and validation cohorts, and both cohorts demonstrated favorable predictive performance, improving the reliability and stability of the model.

In addition, this study excluded severe stage III NEC cases and focused on early stage I to II cases. Therefore, the model is better suited for early NEC screening and aligns with the clinical need for early recognition and early intervention. The ultrasound-based nomogram developed in this study is applicable to preterm infants who meet the following criteria: gestational age < 37 weeks, admission to the NICU within 24 h after birth, and complete clinical and examination data. It is especially suitable for NICU clinicians and ultrasonographers as an auxiliary tool for early NEC screening. For infants assessed as high risk by the model, timely interventions such as temporary cessation of feeding, gastrointestinal decompression, and anti-infective therapy may be implemented to reduce the risk of progression to severe NEC.

However, for preterm infants with congenital gastrointestinal malformations, severe congenital heart disease, or inherited metabolic diseases, the applicability of the model should be evaluated cautiously because these populations were excluded from this study. Direct application of the model to such infants is not recommended.

Although this study achieved meaningful findings, several limitations remain. First, this was a single-center retrospective cohort study, and all participants were recruited from the NICU of our hospital. Geographic factors and differences in medical resources may have introduced selection bias, and the applicability of the model in other regions and hospitals of different levels requires further validation through multicenter studies. Notably, the development cohort yielded approximately 7.5 events per predictor variable, which fell below the conventional EPV threshold of 10 for logistic regression. Second, this study did not include clinical factors such as feeding patterns, intestinal microbiota, or umbilical arterial catheterization, all of which are important contributors to NEC occurrence. As a result, the model may not fully cover all potential risk factors. In addition, the Bell stage I cases included in this study represent suspected NEC with limited diagnostic specificity; diagnostic uncertainty among stage I participants may have affected model estimates. Third, no formal correction for multiple comparisons was applied during univariate screening of risk factors. This raises the possibility of type-I error, and significant associations identified in univariate analyses should be interpreted cautiously. Fourth, the follow-up period was 28 days, which may miss late-onset NEC events occurring beyond this observation window. The predictive performance of the model for long-term NEC occurrence in preterm infants remains unclear. Longer follow-up is needed for further exploration. Fifth, assessment of ultrasound indicators depends on the operator's experience and is therefore somewhat subjective, although acceptable inter-rater reliability was observed in this study. It should be noted that inter-observer reliability testing was only performed on a limited sample of 80 subjects and cannot fully eliminate potential measurement bias in routine clinical practice. Perfusion grades (sparse, absent, hyperperfused) were merged into a binary “abnormal perfusion” variable for nomogram construction; subgroup comparisons across distinct perfusion categories were not pre-specified, and our sample lacked power for such analyses. Larger cohorts are needed to explore the added prognostic value of subdivided perfusion grades. For future research, multi-center external validation across different hospitals is required to confirm generalizability. Further prospective cohort studies are also warranted to verify the real-world predictive efficacy of this non-invasive multimodal nomogram.

Despite these limitations, the present study has important clinical value and scientific significance. From the perspective of clinical application, the ultrasound-based nomogram prediction model is noninvasive, repeatable, and easy to use. By combining readily available laboratory and ultrasound indicators, it can rapidly quantify the risk of early NEC in preterm infants, help clinicians identify high-risk infants at an early stage, and support timely targeted interventions. This may reduce NEC incidence and mortality and improve the prognosis of preterm infants. The model also provides a quantitative basis for NICU decision-making and may reduce missed or incorrect diagnoses caused by insufficient clinical experience.

From the perspective of scientific value, this study systematically analyzed the roles of laboratory inflammatory markers and multiparametric ultrasound indicators in early NEC occurrence, thereby enriching etiological research on early NEC diagnosis. The validated nomogram prediction model provides a standardized research framework for risk prediction in NEC and other critical neonatal diseases. Furthermore, this study confirms the important value of abdominal ultrasound in the early diagnosis of NEC and provides evidence-based support for the broader application of ultrasound technology in diagnosing neonatal gastrointestinal diseases.

In summary, multivariable logistic regression analysis in this study identified gestational age, CRP, PCT, IL-6, bowel wall thickness, decreased intestinal peristalsis, enlarged gastric antral area, decreased gastric antral peristalsis, and abnormal bowel wall blood perfusion as independent predictors of early neonatal NEC. The ultrasound-based nomogram prediction model constructed from these indicators showed good discrimination, calibration, and clinical net benefit after validation in development and validation cohorts. This model provides a quantitative tool for the early, noninvasive clinical diagnosis of NEC, helps facilitate early intervention, improves infant prognosis, and has important significance for the clinical management of critical neonatal disease.

## Data Availability

The original contributions presented in the study are included in the article/Supplementary Material, further inquiries can be directed to the corresponding author.
